# Items

**Published:** 1887-04

**Authors:** 


### ITEMS.

The Twenty-seventh Annual Meeting of the Illinois State Medical Society will be held in the Methodist Church Block, corner Clark and Washington Streets, Chicago, beginning May the 17th, 1887, at 10 o'clock A. M.

INTERNATIONAL MEDICAL CONGRESS.

Professor J. J. Chisolm, M.D., of Baltimore, Md., has been elected President of the Section of Ophthalmology of the Ninth International Medical Congress, in the place of Professor E. Williams, who was compelled to resign on account of ill-health.

Judson B. Andrews, M.D., Superintendent of the Hospital for the Insane, Buffalo, N. Y., has been appointed to the office of President of the Section of Psychological Medicine and Nervous Diseases, made vacant by the recent death of Professor John P. Gray.

No vacancies now exist in the principal offices of the Preliminary Organization of the Congress or of its sections. The latest information regarding all departments is of the most encouraging character.

HOSPITAL APPOINTMENTS.

The annual competitive examinations for the positions of internes at the various Chicago hospitals occurred during the past month, and resulted in the following appointments:

Cook County Hospital—Dr. Buffluer, Dr. J. Hillmantel, Dr. G. J. Kaumheimer, Dr. W. H. Allport, Dr. L. Hektoen,

Dr. C. E. Earle, Dr. A. B. Hart and Dr. Rachel Hickey.

St. Luke's Hospital—Dr. F. Holden and Dr. Edward E. Kerr.

Eye and Ear Infirmary—Dr. G. E. Brinckerhoff.

Michael Reese Hospital—Dr. Stehl and Dr. Greensfielder.

Mercy Hospital—Dr E. E. Kerr and Dr. G. W. Whitfield.

Presbyterian Hospital—Dr. A. J. Mellish and Dr. C. W. Ward.

Woman's Hospital—No appointment.

### COMMENCEMENT EXERCISES AT THE CHICAGO MEDICAL COLLEGES.

At the recent commencement exercises the four regular medical colleges in Chicago have graduated 253 students.

The annual exercises of Rush Medical College were held in Central Music Hall, February 15th, at which time 132 students were graduated.

On February 21st, the College of Physicians and Surgeons held its commencement exercises in the Grand Opera House, and graduated 52 students.

At the annual commencement exercises of the Chicago Medical College (Medical Department of the Nortwestern University), held at Central Music Hall on March 29th, there were 44 graduates.

The Woman's Medical College held its seventeenth annual exercises in the Grand Opera House on April 5th, with a class of 25 graduates.

